# Honokiol Ameliorates Hepatic Lipid Accumulation by Deacetylating PPARG via SIRT3

**DOI:** 10.3390/cells15121095

**Published:** 2026-06-16

**Authors:** Yantao Yang, Shengxiang Guo, Wu Luo, Dongbo Liu, Xincong Kang

**Affiliations:** 1 College of Horticulture, Hunan Agricultural University, Changsha 410128, China; 3275855898@stu.hunau.edu.cn (Y.Y.); samshine0731@outlook.com (S.G.); luowubio@163.com (W.L.); liudongbo@hunau.edu.cn (D.L.); 2 State Key Laboratory of Subhealth Intervention Technology, Hunan Agricultural University, Changsha 410128, China; 3 Hunan Provincial Engineering Research Center of Medical Nutrition Intervention Technology for Metabolic Diseases, Hunan Agricultural University, Changsha 410128, China; 4 National Research Center of Engineering Technology for Utilization of Functional Ingredients from Botanicals, Hunan Agricultural University, Changsha 410128, China

**Keywords:** honokiol, SIRT3, PPARG acetylation, phytomedicine intervention, hepatic lipid accumulation

## Abstract

Dysregulated lipid metabolism is a core pathogenic driver of type 2 diabetes. Honokiol (HKL), the major bioactive constituent of *Magnolia officinalis*, possesses anti-diabetic and lipid-regulatory properties. However, the underlying molecular mechanism remains elusive. This study investigates how HKL ameliorates high-glucose/high-fat (HGHF)-induced hepatic lipid accumulation, with a focus on the role of SIRT3-mediated deacetylation of peroxisome proliferator-activated receptor γ (PPARG). The core targets of HKL were identified through network pharmacology and molecular docking. Human hepatic MIHA cells were treated with glucose (Glu, 40 mM) and palmitic acid (0.2~0.3 mM PA) to establish a lipid accumulation model, followed by treatment with HKL (5–10 μM) with or without a confirmed selective SIRT3 inhibitor 3-(1H-1,2,3-triazol-4-yl) pyridine (3-TYP). Lipid accumulation was assessed by Oil Red O staining and by measuring triglyceride (TG) and total cholesterol (TC) levels. Protein expression and the SIRT3-PPARG interaction were analyzed by Western blot and co-immunoprecipitation (Co-IP). SIRT3 and PPARG were identified as core targets of HKL, exhibiting strong binding with calculated energies of −6.834 and −6.579 kcal/mol, respectively. In MIHA cells, HGHF (40 mM Glu + 0.2–0.3 mM PA) induced lipid accumulation, including increased lipid droplets, and elevated TG (2.5–3.2-fold) and TC (2.2–2.8-fold) contents in a dose-dependent manner, accompanied by downregulated SIRT3/PPARG expression and heightened global protein acetylation. The non-cytotoxic HGHF-M condition (40 mM Glu + 0.2 mM PA) was selected for further experiments. HKL (5–10 μM) dose-dependently reduced lipid accumulation by ~38–60%, decreased TG and TC levels by up to ~13% and ~30%, and restored SIRT3/PPARG expression. The protective effects of HKL were reversed by inhibition of SIRT3 with 3-TYP. Co-IP confirmed the interaction between SIRT3 and PPARG, and SIRT3 overexpression significantly decreased the acetylation level of PPARG. This study suggests that HKL ameliorates hepatic lipid accumulation via SIRT3-mediated deacetylation of PPARG, providing an experimental basis for considering HKL as a potential therapeutic agent against metabolic disorders.

## 1. Introduction

Diabetes mellitus, particularly type 2 diabetes mellitus (T2DM), is a major global health burden, currently affecting approximately 11.11% of the adult population worldwide [1]. Dysregulated lipid metabolism is widely regarded as a core pathogenic driver and critical comorbidity of T2DM. Diabetic dyslipidemia, characterized by mixed hyperlipidemia, is not merely a secondary complication but an integral component of T2DM pathogenesis, forming a reciprocal relationship with insulin resistance [2]. When hepatic lipid accumulation exceeds the liver’s metabolic capacity, lipolysis is enhanced, leading to elevated circulating free fatty acid levels. The accumulation of lipid intermediates, such as diacylglycerols and ceramides, directly impairs insulin signaling cascades [3]. These lipotoxic effects not only promote hepatic gluconeogenesis and reduce peripheral glucose utilization, but also impair pancreatic β-cell function, thereby exacerbating hyperglycemia and worsening the diabetic state. This vicious cycle between dyslipidemia and insulin resistance necessitates the development of interventions that directly disrupt hepatic lipotoxicity. In this context, natural compounds capable of modulating hepatic lipid homeostasis emerge as promising therapeutic strategies for T2DM.

Honokiol (HKL), a major bioactive biphenolic compound from *Magnolia officinalis* barks, has been widely used in traditional Chinese medicine (TCM) to treat thrombotic stroke, anxiety and gastrointestinal symptoms [4]. It exhibits multi-pharmacological effects, including antioxidative, anti-inflammatory, anti-cancer and antimicrobial activity [4,5,6]. Recently, HKL has been reported to exert anti-diabetic activity by enhancing insulin sensitivity and alleviating oxidative stress [7]. HKL lowers blood glucose, suppresses weight gain in diabetic KKAy mice [8], stimulates glucose uptake in muscle and adipocyte cell models [9,10], and scavenges reactive oxygen species to protect pancreatic β-cells under metabolic stress [11]. Importantly, beyond its glucoregulatory actions, HKL also exerts beneficial effects on lipid homeostasis, including decreasing hepatic triglycerides and lipid accumulation [7,12]. However, the mechanism by which HKL regulates lipid metabolism in T2DM remains unclear.

Network pharmacology, which constructs complex “drug–target–pathway–disease” association networks [13,14,15], has provided a key breakthrough in the modernization of TCM research. It aids in exploring drug mechanisms, discovering targets, and systematically understanding multi-component interventions [13]. In target research, network pharmacology identifies core targets from a large number of biomolecules and predicts drug–target interactions, providing a systematic perspective for exploring the potential action mechanisms of TCM [16]. Given its strength in deciphering multi-target mechanisms, network pharmacology is well-suited to identify the potential targets and mode of action of HKL in diabetes intervention.

In this study, network pharmacology was employed to identify the targets and pathways of HKL in diabetes intervention through database mining and network analysis. The results revealed that HKL targets are widely involved in the regulation of glucose and lipid metabolism, with sirtuin3 (SIRT3) and peroxisome proliferator–activated receptor gamma (PPARG) occupying central positions in the interaction network. SIRT3, a mitochondria-resident NAD^+^-dependent deacetylase, is involved in the regulation of hepatic lipid metabolism [17]. It primarily acts by deacetylating key enzymes involved in mitochondrial lipid catabolism [18,19,20]. PPARG, a ligand-activated nuclear transcription factor, is a key regulator of lipid metabolism and an important therapeutic target for T2DM [21]. Its transcriptional activity is dynamically controlled by post-translational modifications, among which acetylation plays an important regulatory role. Hyperacetylation of PPARG inhibits its transcriptional activity, disrupts lipid homeostasis, and promotes lipid accumulation.

However, the specific roles of SIRT3 and PPARG in HKL-mediated lipid metabolism, as well as their potential interplay, remain unclear. Therefore, we integrated molecular docking, co-immunoprecipitation (Co-IP) and functional cell assays to test the hypothesis that HKL targets and binds to SIRT3 to deacetylate PPARG, subsequently regulating lipid metabolism. This work will fill the gap in the mechanism research of HKL in the regulation of lipid metabolism, and promote the translational application of natural compounds in the intervention of metabolic diseases.

## 2. Materials and Methods

### 2.1. Chemicals and Reagents

Honokiol (HKL, CAS No. 35354-74-6, purity ≥ 98%) was purchased from Shanghai Yuanye Biotechnology Co., Ltd. (Shanghai, China). The SIRT3 inhibitor 3-TYP (3-(1H-1,2,3-triazol-4-yl) pyridine) was obtained from Selleck Chemicals (Houston, TX, USA). Dimethyl sulfoxide (DMSO), glucose (Glu), palmitic acid (PA) and bovine serum albumin (BSA) were purchased from Sigma-Aldrich (St. Louis, MO, USA). Dulbecco’s Modified Eagle Medium (DMEM, low-glucose), fetal bovine serum (FBS), penicillin–streptomycin solution, 0.25% trypsin-EDTA, and phosphate-buffered saline (PBS, pH 7.4) were products of Gibco (Thermo Fisher Scientific, Waltham, MA, USA).

Primary antibodies against SIRT3, PPARG, sterol regulatory element-binding protein 1 (SREBP1), glyceraldehyde-3-phosphate dehydrogenase (GAPDH) and horseradish peroxidase (HRP)-conjugated secondary antibodies (goat anti-rabbit IgG; goat anti-mouse IgG) were purchased from Proteintech Group (Wuhan, China). The anti-acetyllysine antibody was obtained from Jingjie Biology (Nanjing, China). The Oil Red O staining kit, triglyceride (TG) assay kit, total cholesterol (TC) assay kit, and BCA protein assay kit were purchased from Nanjing Jiancheng Bioengineering Institute (Nanjing, China). Radio-immunoprecipitation assay (RIPA) lysis buffer and Protein A/G magnetic beads were obtained from Beyotime Biotechnology (Shanghai, China). All other chemicals and solvents were of analytical grade or higher.

### 2.2. Cell Line and Culture Conditions

Human hepatic MIHA cells were purchased from the Cell Bank of the Chinese Academy of Sciences (Shanghai, China). The cells were maintained in DMEM low-glucose medium (Gibco, Carlsbad, CA, USA) supplemented with 10% fetal bovine serum (FBS), 100 U/mL penicillin, and 100 μg/mL streptomycin. Cells were cultured at 37 °C in a humidified incubator with 5% CO_2_, with the medium refreshed every 2–3 days. For subculture, when cells reached 80–90% confluency, the culture monolayer was gently washed twice with pre-warmed PBS (pH 7.4) to remove residual serum. Subsequently, 0.25% trypsin-EDTA solution (Gibco, Carlsbad, CA, USA) was added to the culture flask, and cells were incubated at 37 °C for 2–3 min. Cell digestion was monitored under a microscope. Once the cells rounded up and detached, an equal volume of FBS-containing complete medium was added to neutralize trypsin activity. The cell suspension was then collected and centrifuged at 1000 rpm (approximately 200× *g*) for 3 min at room temperature. The supernatant was discarded, and the cell pellet was resuspended in fresh complete medium. Cells were passaged at a 1:2 split ratio into new culture flasks and then maintained in an incubator.

### 2.3. Prediction of HKL Targets

Potential targets of HKL were retrieved from five databases: GeneCards database (https://www.genecards.org/) (accessed on 15 May 2025), SwissTargetPrediction (http://www.swisstargetprediction.ch/) (accessed on 15 May 2025), SEA (http://sea.bkslab.org/) (accessed on 15 May 2025), STITCH database (http://stitch.embl.de/) (accessed on 15 May 2025), and HERB (http://herb.ac.cn/) (accessed on 15 May 2025), using “honokiol” as the keyword. Only targets shared by at least two independent databases were considered as potential therapeutic targets of HKL. Furthermore, previously reported targets of HKL were collected from the HERB database using “honokiol” as the keyword. Diabetes-associated targets were retrieved from three disease-focused databases: GeneCards (https://www.genecards.org) (accessed on 17 May 2025), DisGeNET (https://www.disgenet.org/) (accessed on 17 May 2025), and CTD (http://ctdbase.org/) (accessed on 17 May 2025). Only targets present in at least two databases were retained as disease-related. Overlapping targets between the HKL-related and diabetes-associated sets were identified using the Venny 2.1.0 online tool (https://bioinfogp.cnb.csic.es/tools/venny/) (accessed on 17 May 2025). Finally, targets from both literature mining and the aforementioned databases were integrated to generate the complete list of HKL-related targets.

### 2.4. Protein–Protein Interaction (PPI) Network and KEGG Pathway Enrichment Analysis

The identified common targets were uploaded to the STRING database (https://string-db.org) (accessed on 10 June 2025) to construct a protein–protein interaction (PPI) network, which was visualized using Cytoscape 3.9.1 software. Node degree values were calculated using the “Network Analyzer” plugin, and the top 10 targets with the highest degree values were identified as key targets. KEGG pathway enrichment analysis was performed using the Metascape platform (https://metascape.org/gp/#/main/step1) (accessed on 15 June 2025) to explore pathways related to lipid metabolism.

### 2.5. Prediction of SIRT3 Acetylation Sites

The full-length amino acid sequence of human SIRT3 (UniProt Accession No.: Q9NTG7) was submitted to the GPS-PAIL 2.0 web server (http://www.pail.biocuckoo.org/) (accessed on 20 June 2025) with the default “medium” threshold. The predicted acetylation sites, including the positions, residues, and confidence scores, were downloaded and filtered. The key predicted acetylation site was then mapped onto the sequence of SIRT3, and the annotated sequence was visualized.

### 2.6. Molecular Docking

The 3D structure of HKL was downloaded as an SDF file from the PubChem database (https://pubchem.ncbi.nlm.nih.gov/) (accessed on 22 May 2025) and converted to PDB format using OpenBabel 3.1.1 (https://openbabel.org/#) (accessed on 25 May 2025). Prior to molecular docking, the compound was processed in AutoDock 1.5.7 (https://ccsb.scripps.edu/projects/docking/) (accessed on 25 May 2025), where water molecules were removed, hydrogen atoms were added, and the compound was designated as the ligand and exported in PDBQT format. The 3D structures of SIRT3 and PPARG were sourced from the RCSB Protein Data Bank (https://www.rcsb.org/) (accessed on 28 May 2025). Docking simulations were performed using AutoDock 1.5.7, with binding energy (≤−5 kcal/mol) considered to indicate interaction affinity. The resulting docking output files were imported into the Protein-Ligand Interaction Profiler to identify key intermolecular interactions, and were visualized using PyMOL 2.6.2 (https://pymol.org/2/) (accessed on 28 May 2025).

### 2.7. Viability Assay

MIHA cells were seeded in 96-well plates at 1 × 10^4^ cells/well and cultured for 24 h to allow adhesion. First, HKL was tested at 0–1280 μM (DMSO ≤ 0.1%) to determine its non-toxic concentration range; then, glucose (Glu, 5.5–200 mM) and palmitic acid (PA, 0.1–2.0 mM in 1% BSA-containing medium) were tested individually to establish safe concentration thresholds. Based on the single-agent results, three high-glucose/high-fat (HGHF) combinations were designed using a fixed non-toxic Glu concentration (40 mM) combined with gradient PA concentrations (0.1, 0.2, and 0.3 mM), designated as HGHF-L, HGHF-M, and HGHF-H, respectively. After 24 h of treatment, 10 μL of CCK-8 reagent was added to each well, followed by 2 h of incubation at 37 °C. Absorbance (OD) was measured at 450 nm using a Tecan Spark microplate reader (Tecan, Männedorf, Switzerland). Relative cell viability was calculated as [(OD_450_ of treated group − OD_450_ of blank control)/(OD_450_ of cell control − OD_450_ of blank control)] × 100%. Each group consisted of 6 replicate wells, and the experiment was repeated three times independently. Non-toxic concentrations of HKL and HGHF were selected for subsequent experiments.

### 2.8. Oil Red O Staining

Intracellular lipid droplets were visualized by Oil Red O staining using a commercial kit according to the manufacturer’s instructions. Briefly, cells were washed twice with ice-cold PBS and fixed with 4% paraformaldehyde for 30 min at room temperature, and then stained with the kit’s Oil Red O reagents. Nuclei were counterstained with hematoxylin. Images were acquired using a Leica DMi8 inverted microscope at 20× objective magnification, with identical light intensity and exposure settings applied across all groups. At least five random fields per group were captured, and representative images are shown. For semi-quantitative analysis, the mean optical density (MOD) of Oil Red O-positive lipid droplets was measured using ImageJ software 1.54 (https://imagej.net/), normalized to the total cell area per field. All data were expressed as a percentage of the control group (set as 100%). Results were analyzed and visualized using GraphPad Prism 9.5.0 (GraphPad Software, Boston, MA, USA).

### 2.9. TG/TC Measurement

After treatment, cells were washed twice with pre-chilled PBS to remove residual medium. Cells were then lysed in ice-cold lysis buffer (0.1% Triton X-100 in PBS) on ice for 30 min to ensure complete lysis. The lysates were centrifuged at 12,000 rpm for 10 min at 4 °C, and the supernatants were collected. Intracellular triglyceride (TG) and total cholesterol (TC) levels were measured using commercial assay kits (Nanjing Jiancheng Bioengineering Institute, Nanjing, China) according to the manufacturer’s instructions, with absorbance read at 500 nm. The measured values were normalized to the total protein concentration, determined using a BCA protein assay kit (Tiangen Biotech, Beijing, China) to correct for differences in cell number and lysis efficiency. All data were normalized to the control group and expressed as percentages of control.

### 2.10. SIRT3 Overexpression

Total RNA was isolated from wild-type human hepatic MIHA cells. The full-length coding sequence of human SIRT3 was amplified by RT-qPCR using gene-specific primers. The PCR product was separated by agarose gel electrophoresis, purified and inserted into the linearized pCDH-CMV-MCS-EF1-GFP + Puro lentiviral vector via homologous recombination to generate the recombinant plasmid pCDH-SIRT3. For transfection, the plasmid pCDH-SIRT3 DNA was introduced into 293T packaging cells using the Calcium Phosphate Transfection Kit (Beyotime Biotechnology, Shanghai, China) according to the manufacturer’s instructions. Briefly, the plasmid DNA was mixed with the calcium phosphate reagent to form a DNA–calcium phosphate precipitate, which was then added to the cell culture medium. After 48 h, the viral supernatant was collected, filtered through a 0.45 μm filter, and used to transduce MIHA cells in the presence of 8 μg/mL polybrene. Following transduction, stable SIRT3-overexpressing MIHA cell lines were selected by culturing in medium containing 2 μg/mL puromycin for 7–10 days. Overexpression efficiency was verified by Western blotting and RT-qPCR.

### 2.11. Western Blotting

Treated MIHA cells were washed twice with ice-cold PBS (pH 7.4) to remove residual medium. For total protein extraction, cells were lysed in ice-cold RIPA lysis buffer supplemented with protease and phosphatase inhibitors. Protein concentrations were determined using a BCA protein assay kit. Equal amounts of protein were mixed with 5× SDS loading buffer and denatured at 100 °C for 5 min. Proteins were then separated by sodium dodecyl sulfate-polyacrylamide gel electrophoresis and transferred to polyvinylidene difluoride (PVDF) membranes using a wet transfer system (Tanon, shanghai, China) at constant current of 200 mA for 50 min on ice. The membranes were blocked with 5% non-fat milk in TBST (Tris-Buffered Saline with Tween 20) buffer for 1 h at room temperature. The membranes were incubated overnight at 4 °C with primary antibodies against SIRT3 (1:5000) and PPARG (1:3000). After washing three times with TBST, the membranes were incubated with corresponding HRP-conjugated secondary antibodies for 1 h at room temperature. Immunoreactive bands were visualized using an enhanced chemiluminescence reagent and captured with a chemiluminescence imaging system. Band intensities were quantified using ImageJ software 1.54, and protein expression levels were normalized to GAPDH or tubulin as the internal control.

### 2.12. Co-Immunoprecipitation (Co-IP)

Cells were lysed in ice-cold IP lysis buffer supplemented with protease and deacetylase inhibitors to preserve protein–protein interaction and acetylation status. Lysates were centrifuged at 12,000× *g* for 15 min at 4 °C, and the supernatants were collected. For immunoprecipitation, 5 μg of anti-SIRT3 antibody was added to the supernatants, followed by incubation with Protein A/G magnetic beads (Beyotime) at 4 °C for 4 h with gentle rotation. The beads were then washed three times with ice-cold IP buffer to remove non-specific bindings. The immunoprecipitated complexes were eluted by boiling in 2× SDS loading buffer and analyzed by Western blotting using an anti-PPARG or anti-SIRT3 antibody.

### 2.13. Statistical Analysis

Data are presented as mean ± standard deviation (SD). Normality was assessed using the Shapiro–Wilk test, and homogeneity of variances was evaluated using Levene’s test in GraphPad Prism 9.5.0. After confirming that the data met both assumptions, parametric tests were applied. Statistical significance for multiple group comparisons was determined by one-way ANOVA followed by Tukey’s post hoc test. A *p*-value < 0.05 was considered statistically significant.

## 3. Results

### 3.1. Network Association of HKL Targeting SIRT3 and PPARG

To identify potential molecular targets of HKL for the intervention of hepatic lipid metabolism, network pharmacology analysis and molecular docking were performed. A total of 269 HKL targets were identified from five databases—GeneCards, Swiss Target Prediction, SEA, STITCH database and HERB—of which 28 targets appeared in at least two databases. Additionally, 71 HKL targets reported in the literature were retrieved from the HERB database. After removing duplicates, these were integrated with the aforementioned 28 targets, resulting in a total of 91 therapeutic targets for HKL. From three disease databases—GeneCards, DisGeNET, and CTD—we identified a total of 1558 disease targets appearing in at least two databases. The HKL and diabetes target sets were analyzed using the Venny 2.1.0 online tool, revealing 72 common HKL-diabetes targets (Figure S1A).

**Figure 1:**
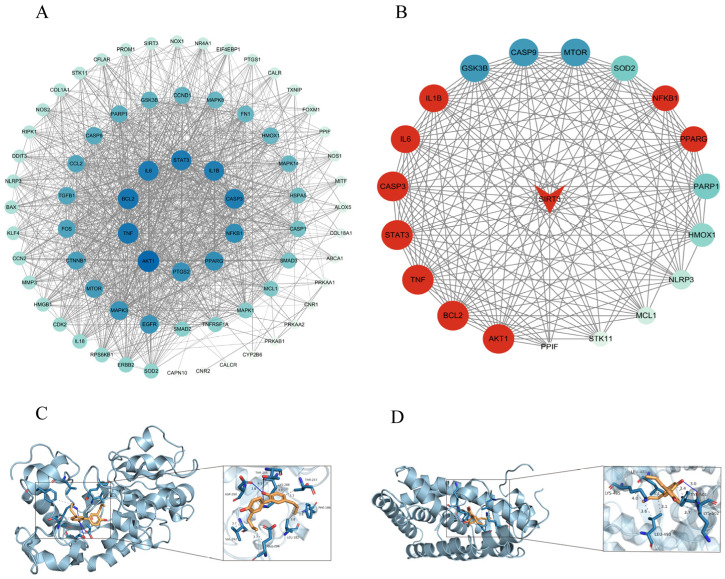
Identification of core targets and pathways of HKL in ameliorating hepatic lipid accumulation. (**A**) Protein–protein interaction (PPI) network of the 72 common targets constructed using the STRING database and visualized by Cytoscape 3.9.1. Nodes denote target proteins, and edges represent protein–protein interaction. (**B**) Top 10 key targets ranked by degree value in the PPI network, including peroxisome proliferator-activated receptor γ (PPARG) and sirtuin 3 (SIRT3), which are core regulators of lipid metabolism and diabetes. (**C**,**D**) Molecular docking models of HKL with SIRT3 (**C**) and PPARG (**D**). HKL (green) binds to the active pocket of SIRT3 (red) via hydrogen bonds and hydrophobic interactions (binding energy: −6.834 kcal/mol) (**C**), and interacts with PPARG (blue) through hydrophobic interactions (binding energy: −6.579 kcal/mol) (**D**). Binding energies below −5.0 kcal/mol indicate strong affinity between HKL and the two targets. All data are derived from three independent database analyses or docking simulations.

To link drug targets with disease targets and elucidate the potential pharmacological mechanism by which HKL improves lipid metabolism and hyperlipidemia, we used the merge function in Cytoscape 3.10. The resulting network included 72 nodes and 1129 interaction edges. The 72 candidate targets were ranked by degree value, and the top 10 targets (the highest degree values) were considered as key targets and placed centrally (Figure 1A,B). KEGG enrichment analysis of the 72 targets revealed the top 40 enriched pathways (Figure S1B), among which “Lipid and atherosclerosis” was the most significant (smallest *p*-value), suggesting that HKL may effectively regulate lipid metabolism.

Among the 72 candidate targets, PPARG, a nuclear receptor that governs lipid homeostasis, was ranked within the top 10. Its transcriptional activity is known to be regulated by deacetylation modification [22,23]. SIRT3, a mitochondrial deacetylase, was also identified among the 72 HKL targets for T2DM intervention. In the PPI network, SIRT3 was found to interact with PPARG and eight other key targets within the top 10.

Molecular docking was performed using AutoDock Vina 1.5.7 to assess the binding of HKL to SIRT3 and PPARG. HKL bound to SIRT3 through hydrogen bonds and hydrophobic interactions, with a binding energy of −6.834 kcal/mol. It bound to PPARG via hydrophobic interactions, with a binding energy of −6.579 kcal/mol (Figure 1C,D). Both binding energies were below −5.0 kcal/mol, indicating strong interactions between HKL and these two proteins.

### 3.2. Reduced SIRT3 Expression and Increased Acetylation in HGHF-Induced Lipid-Accumulated Hepatic Cells

**Figure 2:**
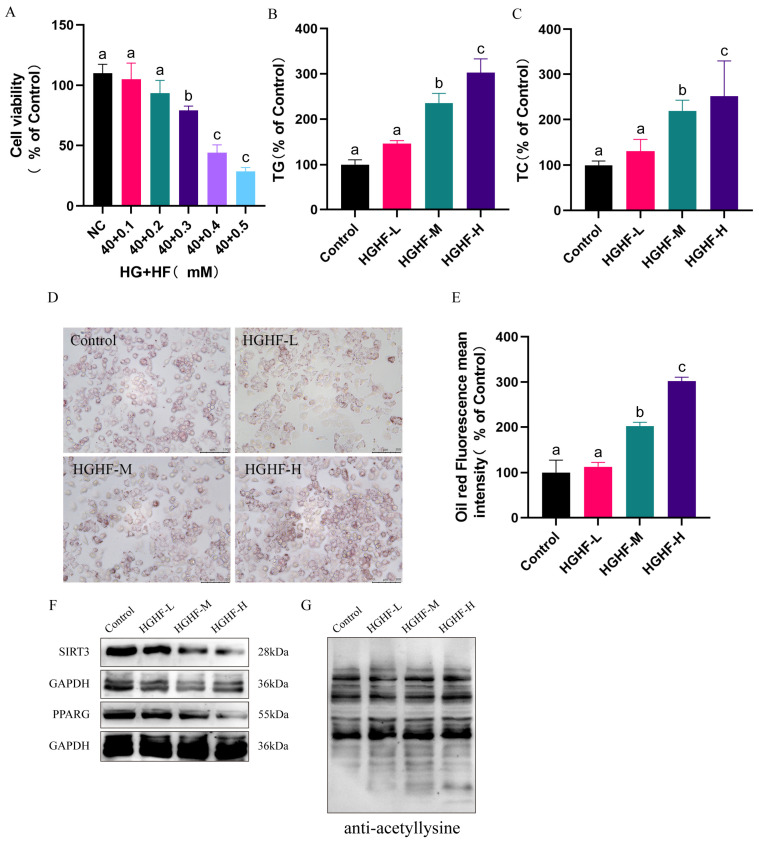
Establishment of high-glucose/high-fat (HGHF)-induced hepatic lipid accumulation model and alterations in SIRT3/PPARG expression and global acetylation levels. (**A**) Cell viability of MIHA cells after 24 h treatment with different HGHF combinations (40 mM glucose + 0.1–0.5 mM PA). Cell viability was significantly reduced at PA ≥ 0.3 mM. Data are presented as mean ± SD (*n* = 3). Different letters (a, b, c) indicate significant differences between groups (*p* < 0.05). (**B**,**C**) Triglyceride (TG) and total cholesterol (TC) content in MIHA cells after HGHF treatment. Data are presented as mean ± SD (*n* = 3). Statistical letters indicate significant differences among groups (one-way ANOVA with Tukey’s post hoc test, *p* < 0.05). (**D**) Representative Oil Red O staining images of lipid droplets (red) in MIHA cells. Scale bar = 100 µm. (**E**) Semi-quantitative analysis of Oil Red O staining intensity measured using ImageJ 1.54 and normalized to the control group. Data are expressed as percentage of control (mean ± SD, *n* = 5 fields per condition). Statistical letters indicate significant differences among groups (one-way ANOVA with Tukey’s post hoc test, *p* < 0.05). (**F**) Western blot analysis of SIRT3 (28 kDa) and PPARG (55 kDa) protein expression. GAPDH (36 kDa) served as internal reference for normalization. The membrane was cut into strips prior to incubation with individual antibodies. (**G**) Western blot analysis of global protein acetylation levels detected by anti-acetyllysine antibody.

To establish a HGHF-induced lipid accumulation model in MIHA cells, we first assessed cell viability after treatment with increasing concentrations of Glu or PA alone. Cell viability remained unaffected at Glu ≤ 40 mM or PA ≤ 0.2 mM (Figure S2A,B). We next evaluated cell viability under combined treatment with 40 mM Glu (HG) and increasing PA concentrations (0.1–0.5 mM, HF). Viability was not affected at PA ≤ 0.2 mM but declined at 0.3 mM by approximately 21% (*p* < 0.05 vs. Control, Figure 2A), with further reductions of 55% and 70% at 0.4 mM and 0.5 mM, respectively (*p* < 0.05 vs. Control, Figure 2A).

Compared to the control group, TG levels in the HGHF-M and HGHF-H groups increased by approximately 2.5-fold and 3.2-fold, respectively (both *p* < 0.01, Figure 2B), while TC levels rose by approximately 2.2-fold and ~2.8-fold, respectively (both *p* < 0.01, Figure 2C). Oil Red O staining results indicated a dose-dependent increase in lipid droplet formation (Figure 2D,E).

To further clarify the roles of SIRT3 and PPARG in lipid metabolism regulation, we examined the protein expression of SIRT3 and PPARG, as well as global protein acetylation levels, in the HGHF-induced cellular model. Both SIRT3 and PPARG protein expressions were significantly downregulated in a dose-dependent manner following HGHF treatment (Figure 2F), while global protein acetylation levels were notably elevated (Figure 2G). Together, these results indicate that HGHF treatment induces lipid accumulation in MIHA cells, accompanied by reduced expression of SIRT3 and PPARG and increased global acetylation. Based on these findings, the HGHF-M condition (40 mM Glu + 0.2 mM PA) was selected for subsequent experiments, as it induced robust lipid accumulation and hyperacetylation without significant cytotoxicity.

### 3.3. Honokiol (HKL) Ameliorates Hepatic Lipid Accumulation in a Dose-Dependent Manner

The viability of MIHA cells remained unaffected at HKL concentrations up to 40 μM, but decreased markedly at 80 μM or higher (Figure S3). HKL has been reported to ameliorate hepatic steatosis in mice at a dietary dose of 0.02% (*w*/*w*) [7]. Based on its pharmacokinetic profile [24] and previous in vitro studies [25], the concentration range of 5–10 µM was selected for subsequent experiments, designated as Treat-5 and Treat-10.

**Figure 3:**
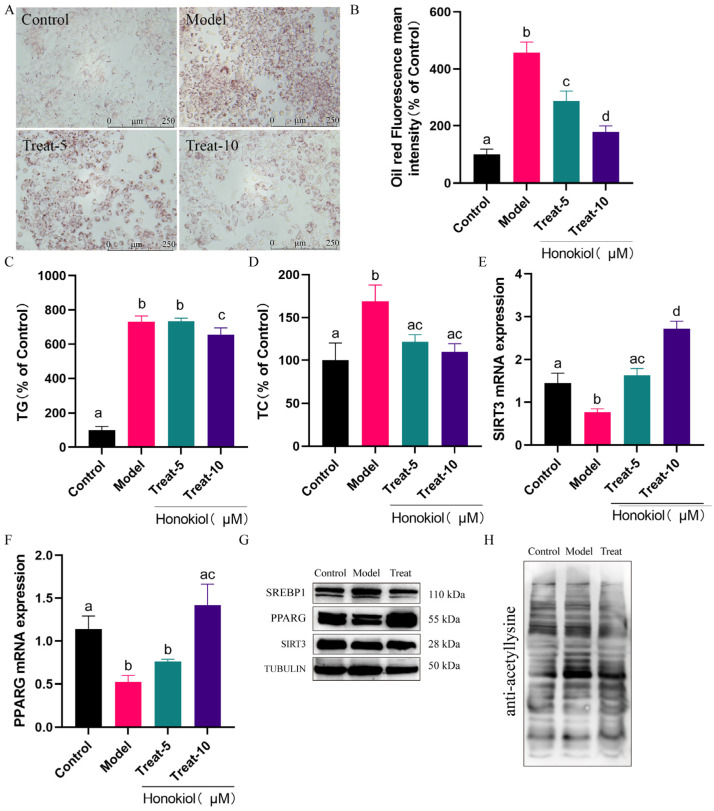
Honokiol (HKL) ameliorates HGHF-induced hepatic lipid accumulation in a dose-dependent manner. (**A**) Representative Oil Red O staining images of lipid droplets (red) in MIHA cells. Scale bar = 250 µm. (**B**) Semi-quantitative analysis of Oil Red O staining intensity measured using ImageJ 1.54 and normalized to the control group. Data are expressed as percentage of control (mean ± SD, *n* = 5 fields per condition). (**C**,**D**) TG and TC content in MIHA cells after HKL treatment. Data are presented as mean ± SD (*n* = 3). Statistical letters indicate significant differences among groups (one-way ANOVA with Tukey’s post hoc test, *p* < 0.05). (**E**,**F**) Relative mRNA expression levels of SIRT3 (**E**) and PPARG (**F**) detected by RT-qPCR. Data were normalized to GAPDH and are presented as mean ± SD from three independent experiments (*n* = 3). Statistical letters indicate significant differences among groups (one-way ANOVA with Tukey’s post hoc test, *p* < 0.05). (**G**,**H**) Western blot analysis of PPARG, SIRT3 protein expression (**G**) and global acetylation levels (**H**) in control, model, and HKL-treated groups. The membrane was cut into strips prior to incubation with individual antibodies in (**G**).

Oil Red O staining demonstrated a dose-dependent reduction in lipid droplet accumulation upon HKL treatment, with decreases of approximately 38% (*p* < 0.01) and 60% (*p* < 0.01) in the Treat-5 and Treat-10 groups, respectively (Figure 3A,B). Consistently, HKL treatment dose-dependently reversed these lipid disturbances. Specifically, in the Treat-10 group, TG levels were reduced by approximately 13% (*p* < 0.01), and TC levels by approximately 35% (*p* < 0.01), respectively (Figure 3C,D).

HKL treatment also dose-dependently restored the mRNA expression of SIRT3 and PPARG. In the Treat-10 group, SIRT3 mRNA levels increased by approximately 2.32-fold (*p* < 0.01) and PPARG mRNA levels by approximately 1.73-fold (*p* < 0.01, Figure 3E,F). These changes were further confirmed at the protein level by Western blotting (Figure 3G). Compared to the HGHF group, HKL treatment (10 µM) restored SIRT3 and PPARG protein expression. In line with this, HKL treatment also attenuated global protein acetylation, consistent with the restored activity of the deacetylase SIRT3 (Figure 3H). Additionally, HKL treatment downregulated the expression of the lipogenic transcription factor SREBP1, which plays a key role in hepatic lipid synthesis, consistent with the reduction in lipid accumulation. Together, these findings demonstrate that HKL ameliorates HGHF-induced hepatic lipid accumulation, restores SIRT3/PPARG expression and reduces protein acetylation in MIHA cells.

### 3.4. Honokiol Ameliorates Lipid Accumulation in a SIRT3-Dependent Manner

**Figure 4:**
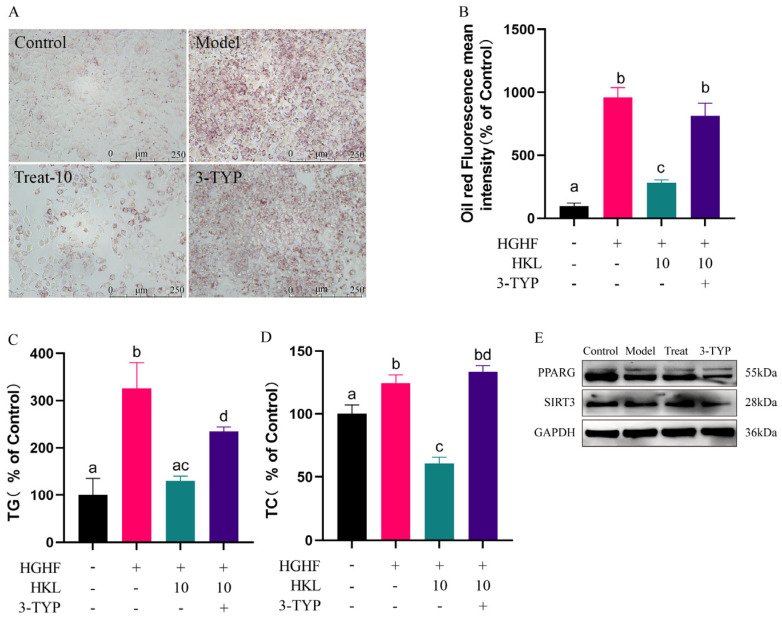
Honokiol (HKL) ameliorates lipid accumulation in an SIRT3-dependent manner. (**A**) Representative Oil Red O staining of lipid droplets (red) in control, model, Treat-10 (10 μM HKL), and 3-TYP-treated groups. Scale bar = 250 µm. (**B**) Semi-quantitative analysis of Oil Red O staining intensity measured using ImageJ 1.54 and normalized to the control group. Data are expressed as percentage of control (mean ± SD, *n* = 5 fields per condition). (**C**,**D**) Triglyceride (TG) and total cholesterol (TC) levels in control, model, Treat-10, and 3-TYP-treated groups. Data are presented as mean ± SD (*n* = 3). Statistical letters indicate significant differences among groups (one-way ANOVA with Tukey’s post hoc test, *p* < 0.05). (**E**) Western blot analysis of proteins SREBP1 (110 kDa), PPARG (55 kDa) and SIRT3 (28 kDa), with GAPDH (36 kDa) as the internal loading control. The membrane was cut into strips prior to incubation with individual antibodies.

To determine whether the lipid-lowering effect of HKL is mediated through SIRT3, we co-treated HGHF-exposed MIHA cells with HKL (10 μM) and the specific SIRT3 inhibitor 3-TYP. Compared with the HKL-only group, the addition of 3-TYP significantly attenuated the protective effects of HKL (Figure 4A–D). Specifically, 3-TYP co-treatment led to a marked increase in lipid droplet deposition (approximately 2.14-fold, *p* < 0.01), intracellular TG levels (approximately 85%, *p* < 0.01) and TC levels (approximately 1.24-fold, *p* < 0.01). In parallel, 3-TYP co-treatment downregulated SIRT3 and PPARG protein expression, while upregulating SREBP1 expression (Figure 4A–E). These results indicate that inhibition of SIRT3 largely abolishes the beneficial effects of HKL on HGHF-induced lipid accumulation, suggesting that the lipid-ameliorating activity of HKL is, at least in part, dependent on SIRT3.

### 3.5. HKL Ameliorates PPARG Acetylation via Direct SIRT3-PPARG Interaction

**Figure 5:**
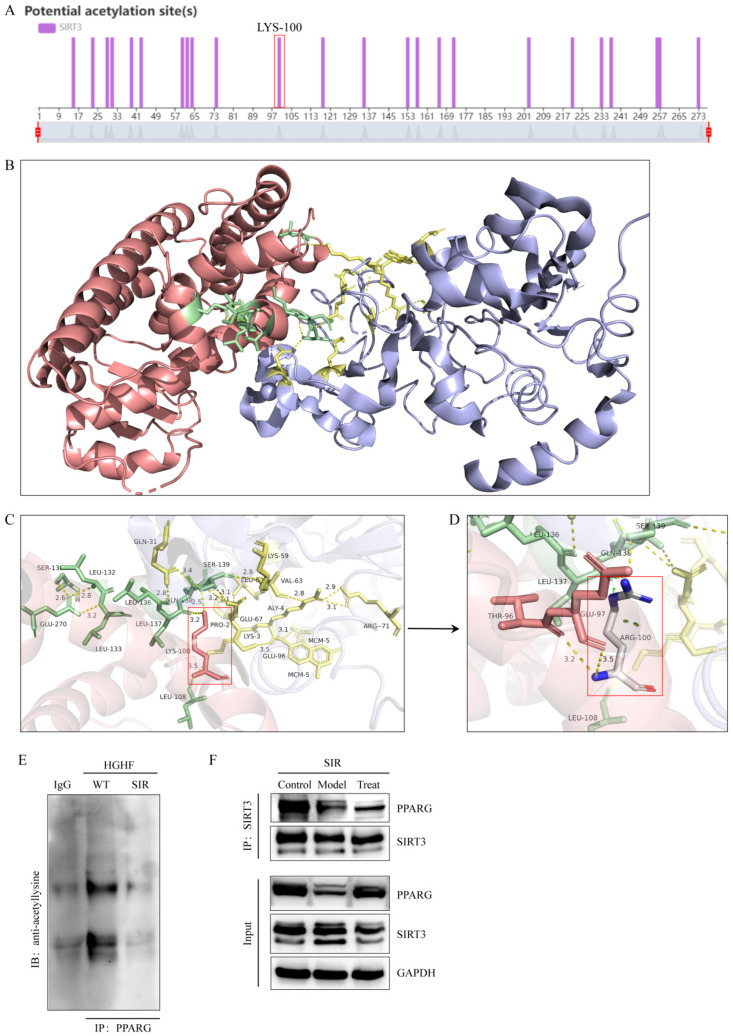
SIRT3 acetylation site prediction, HKL-SIRT3-PPARG binding, and SIRT3-PPARG interaction. (**A**) Prediction of SIRT3 acetylation sites via GPS-PAIL 2.0. Lys-100 (marked) is identified as a key acetylation site. (**B**) Molecular docking model of the HKL-SIRT3-PPARG complex. SIRT3 (red), PPARG (blue), and HKL (green); HKL stabilizes the SIRT3-PPARG interaction. (**C**,**D**) HKL-SIRT3 binding interactions: (**C**) Overall binding mode; (**D**) Local interactions between HKL and SIRT3 (Lys-100 region). Hydrogen bonds are shown as dashed lines, which enhance complex stability. (**E**) SIRT3 overexpression (SIR) reduces PPARG acetylation compared with wild-type (WT); IP: PPARG; immunoblot (IB): anti-acetyllysine; IgG: negative control. (**F**) HKL restores the SIRT3-PPARG interaction in the HKL-treated (Treat) group compared with the HGHF-treated (Model) group. IP: SIRT3; IB: PPARG; Input: total protein loading control. The membrane was cut into strips prior to incubation with individual antibodies.

To identify potential molecular targets of HKL, we performed in silico analysis to predict candidate regulatory sites of SIRT3. GPS-PAIL 2.0 prediction identified Lys-100 as a candidate acetylation site for SIRT3 (Figure 5A). Molecular docking was then performed to visualize the direct interaction between HKL and SIRT3 and its potential effect on SIRT3-PPARG complex formation. As shown in Figure 5B, HKL (green) bound specifically to the active pocket of SIRT3 (red), stabilizing its conformation. Detailed binding interaction analysis (Figure 5C,D) revealed that HKL formed multiple hydrogen bonds and hydrophobic contacts with SIRT3, which may enhance SIRT3’s capacity to interact with PPARG (blue, Figure 5B). These findings suggest that HKL might stabilize the SIRT3-PPARG complex.

Co-IP was performed to investigate the relationship between SIRT3 and PPARG acetylation. Wild-type (WT) and SIRT3-overexpressing (SIR) MIHA cells were treated with HGHF, followed by immunoprecipitation using an anti-PPARG antibody (rabbit) and Western blotting with an anti-acetyllysine antibody (mouse). SIRT3 overexpression significantly reduced the acetylation level of PPARG (Figure 5E). To confirm the direct interaction between SIRT3 and PPARG during HKL treatment, we conducted Co-IP assay in control, HGHF-treated (Model), and HKL-treated (Treat) groups using SIRT3-overexpressing MIHA cells. As shown in Figure 5F, a specific band corresponding to PPARG was detected in anti-SIRT3 immunoprecipitates, indicating an interaction between SIRT3 and PPARG. Notably, under HGHF conditions, SIRT3 expression decreased, and the amount of PPARG co-precipitated with SIRT3 was significantly reduced. HKL treatment restored this interaction (Figure 5F).

## 4. Discussion

Hepatic lipid accumulation is a key pathogenic factor in T2DM, and deciphering the molecular regulators of lipid homeostasis is crucial for developing therapeutic strategies. Here, we provided evidence that HKL, a bioactive compound from *Magnolia* species, mitigates HGHF-induced hepatic lipid accumulation by targeting the SIRT3-PPARG axis, with implications for combating lipid-related metabolic diseases.

SIRT3 deacetylates key enzymes involved in mitochondrial lipid catabolism—including long-chain and medium-chain acyl-CoA dehydrogenase—thereby enhancing fatty acid oxidation and maintaining lipid homeostasis [18,19,26]. *Sirt3*-knockout mice fed a high-fat diet exhibit exacerbated hepatic steatosis, attributed to hyperacetylation and functional impairment of lipid metabolic enzymes [27]. Conversely, SIRT3 overexpression mitigates lipotoxicity in hepatocytes under lipid-overloaded conditions [28]. In this study, HKL treatment promoted SIRT3 expression, whereas co-treatment with the SIRT3 inhibitor 3-TYP abolished the ameliorative effects of HKL on TG, TC, and Oil Red O staining. These findings indicate that HKL improves lipid metabolism by targeting SIRT3, consistent with previous reports identifying HKL as a SIRT3 activator [29].

To further elucidate the mechanism of action of SIRT3, we performed SIRT3 overexpression, which resulted in reduced PPARG acetylation. PPARG governs hepatic lipid metabolic balance by transcriptionally regulating genes involved in lipid uptake, lipogenesis, and fatty acid oxidation [30]. Its transcriptional activity is regulated by acetylation. Hyperacetylation impairs PPARG function, disrupting lipid homeostasis and promoting lipid deposition, whereas deacetylation restores its regulatory activity [30]. PPARG is acetylated by p300 or CBP on multiple lysine residues, and plays an important role in lipid synthesis [31]. Deacetylation at K268 and K293 by SIRT1 or SIRT7 has been shown to selectively activate lipid oxidative genes (e.g., *cpt1a*) while repressing lipogenic genes (e.g., *Srebp1c*, *Acaca*, *Fasn*, etc.) [32,33,34]. Our study demonstrates that SIRT3-mediated deacetylation of PPARG exerts a lipid-lowering effect by inhibiting SREBP1-driven lipogenesis, as evidenced by reduced TG, TC, and Oil Red O staining. Notably, in silico prediction identified Lys-100 as a putative SIRT3-targeted deacetylation site, a finding that awaits experimental validation.

The interaction between SIRT3 and PPARG was confirmed by Co-IP in HKL-treated cells, consistent with previous reports in other biological contexts. In cardiac remodeling, SIRT3 regulates PPARG-mediated cardiolipin biosynthesis to maintain the mitochondrial structure and function [35]. In cardiac fibrosis, SIRT3 prevents cardiac fibroblast transdifferentiation by upregulating PPARG expression [36]. Conversely, PPARG has also been reported to activate SIRT3 [37]. In our study, an interaction between SIRT3 and PPARG was observed, with SIRT3 regulating PPARG expression. However, whether PPARG reciprocally regulates SIRT3 expression remains to be explored.

Several limitations of this study should be acknowledged. First, the lack of validation in primary hepatocytes or in vivo models limits the physiological relevance of our findings. Second, the proposed deacetylation of PPARG at Lys-100 by SIRT3 is based on in silico prediction and requires experimental confirmation, such as site-directed mutagenesis. Third, although Co-IP confirmed an interaction between SIRT3 and PPARG, whether PPARG reciprocally regulates SIRT3 expression remains unknown. Future studies addressing these limitations will be essential to fully elucidate the SIRT3-PPARG axis in hepatic lipid metabolism.

## 5. Conclusions

In summary, HKL alleviates HGHF-induced hepatic lipid accumulation by promoting SIRT3-mediated deacetylation of PPARG, thereby restoring its normal lipid-regulatory function. This study suggests a novel molecular mechanism for HKL’s lipid-lowering effect and indicates that SIRT3 acts as an upstream deacetylase of PPARG in hepatocytes, a hypothesis that awaits further testing. The findings provide an experimental basis for developing HKL as a potential therapeutic agent for metabolic disorders.

## Data Availability

The original contributions presented in this study are included in the article/Supplementary Materials. Further inquiries can be directed to the corresponding author.

## Supplementary Materials

The following supporting information can be downloaded at: https://www.mdpi.com/article/10.3390/cells15121095/s1, Figure S1: Network pharmacology-based prediction of honokiol (HKL) targets and pathways in hepatic lipid metabolism disorders. (A) Venn diagram showing the overlap between honokiol-related targets and disease-related targets. (B) KEGG pathway enrichment analysis of the shared targets. Figure S2: Effects of glucose (A) and palmitic acid (PA) (B) on MIHA cell viability. Figure S3: Effects of honokiol (HKL) on MIHA cell viability.

## Abbreviations

3-TYP, 3-(1H-1,2,3-triazol-4-yl) pyridine; BSA, Bovine serum albumin; Glu, Glucose; HGHF, High-glucose/high-fat; HGHF-L, High-glucose/high-fat-low; HGHF-M, High-glucose/high-fat-medium; HGHF-H, High-glucose/high-fat-high; HKL, Honokiol; PA, Palmic acid; TC, Total Cholesterol; TG, Triglycerides; Treat-5, Treatment with 5 μM HKL; Treat-10, Treatment with 10 μM HKL.
