# Supplementary figures and images for: Honokiol Ameliorates Hepatic Lipid Accumulation by Deacetylating PPARG via SIRT3
Source: Cells. 2026 Jun 16;15(12):1095. doi: 10.3390/cells15121095 (PMC13297477; doi:10.3390/cells15121095)

Figure S1

A

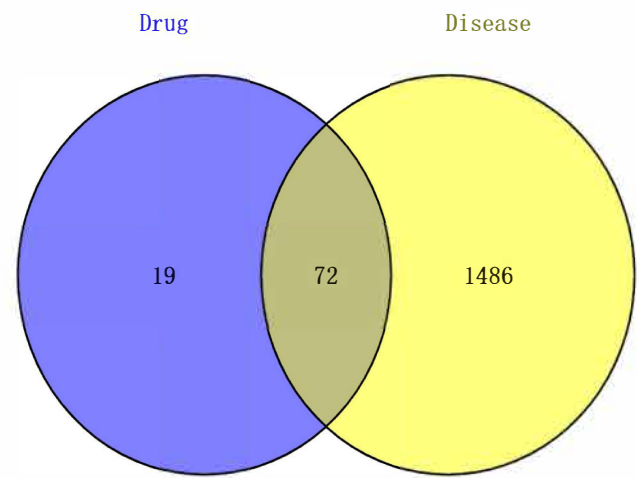

B

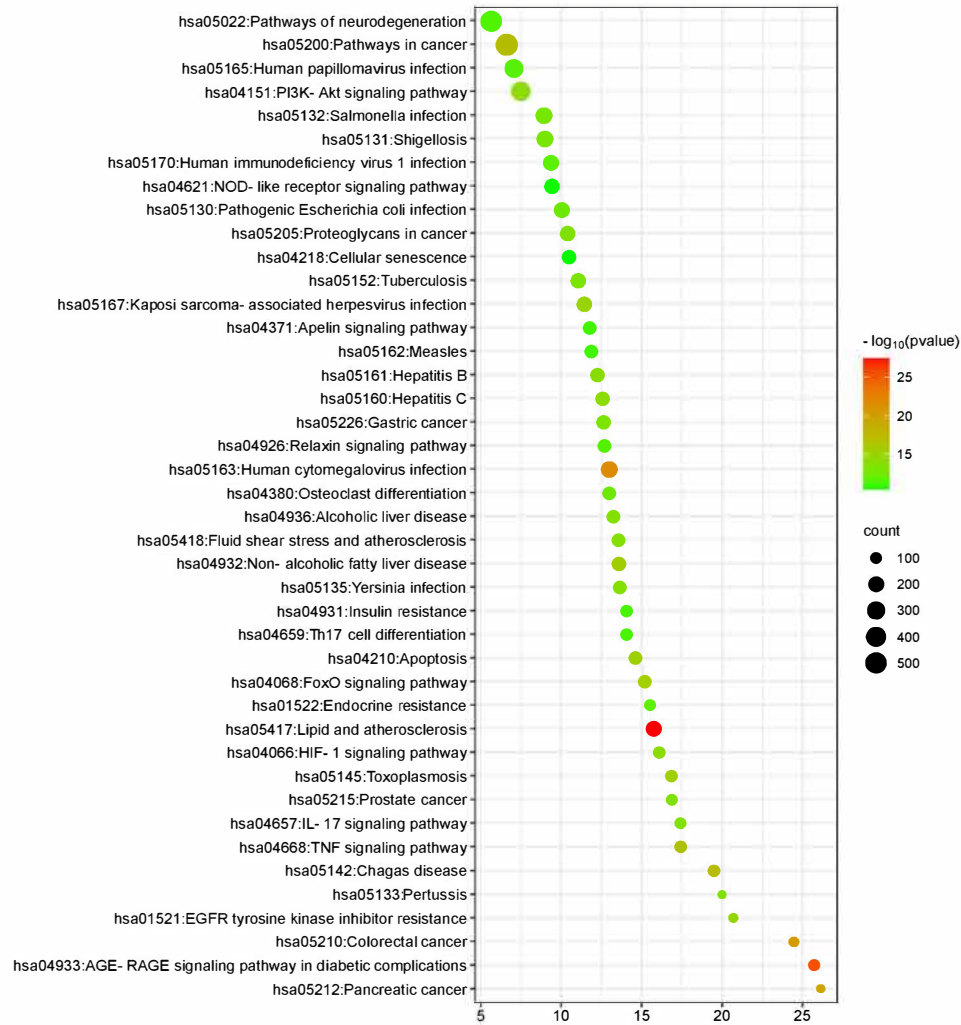

Figure S2

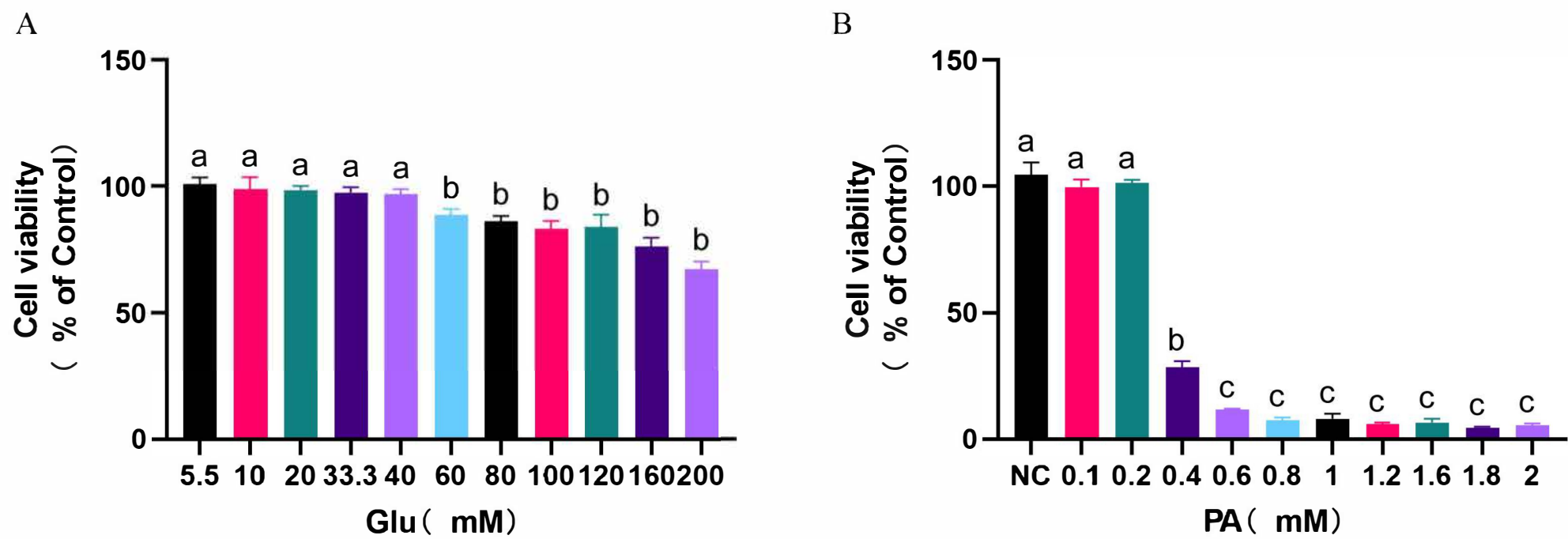

Figure S3

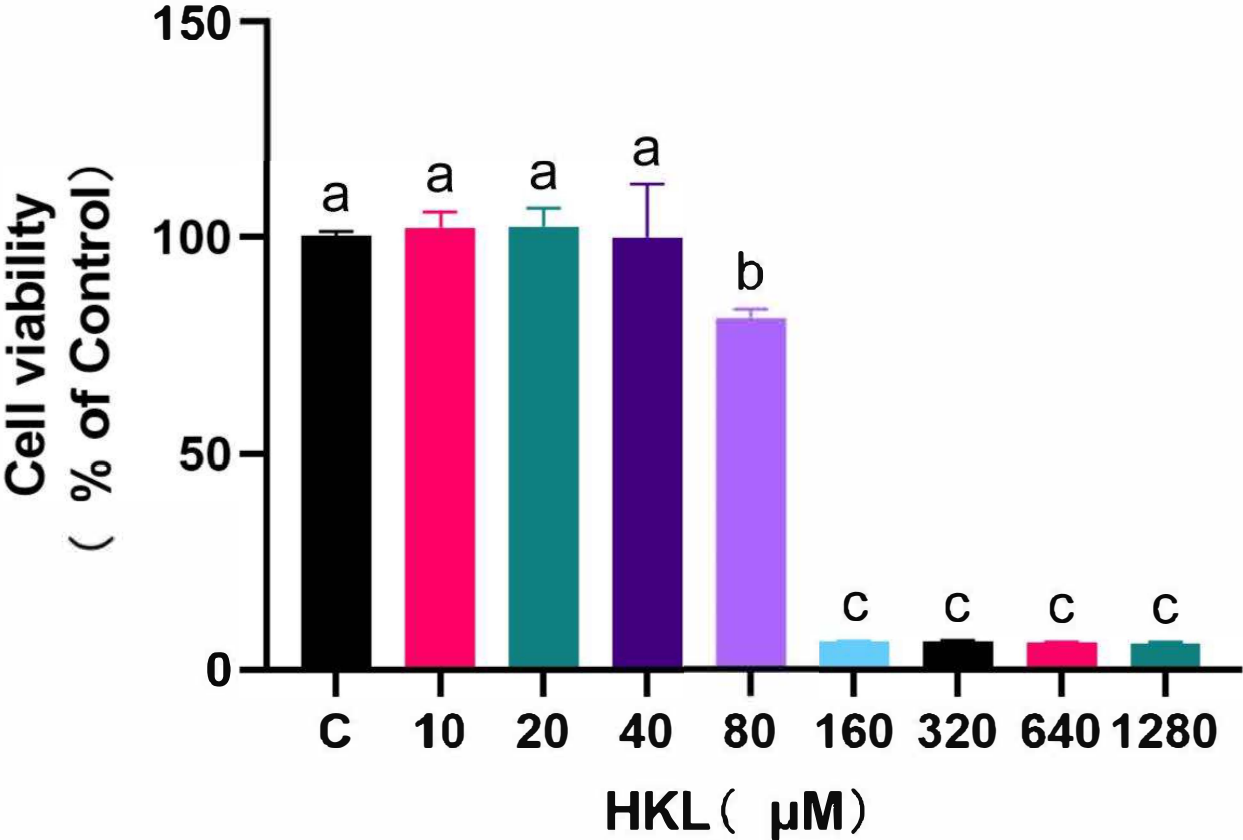

Supplement: Supplementary file 1 [file cells-15-01095-s001.zip › cells-4292545-supplementary.pdf]
